# Pre- and postdiagnosis growth failure, adult short stature, and untreated growth hormone deficiency in radiotherapy-treated long-term survivors of childhood brain tumor

**DOI:** 10.1371/journal.pone.0274274

**Published:** 2022-09-06

**Authors:** Julia Anttonen, Tiina Remes, Pekka Arikoski, Päivi Lähteenmäki, Mikko Arola, Arja Harila-Saari, Tuula Lönnqvist, Tytti Pokka, Pekka Riikonen, Kirsti Sirkiä, Heikki Rantala, Marja Ojaniemi

**Affiliations:** 1 Department of Pediatrics and Adolescence, PEDEGO Research Unit and Medical Research Center, University and University Hospital of Oulu, Oulu, Finland; 2 Department of Pediatrics and Adolescence, Helsinki University Hospital, Helsinki, Finland; 3 Kuopio Pediatric Research Unit, University of Eastern Finland and Kuopio University Hospital, Kuopio, Finland; 4 Department of Pediatrics and Adolescent Medicine, Turku University Hospital and Turku University, Turku, Finland; 5 Faculty of Medicine and Life Sciences, Department of Pediatrics, Tampere University Hospital and University of Tampere, Tampere, Finland; 6 Department of Women’s and Children’s Health, Uppsala University, Uppsala, Sweden; Danish Cancer Society, DENMARK

## Abstract

**Purpose:**

Growth failure is common in radiotherapy-treated long-term survivors of pediatric brain tumors, but studies on longitudinal growth in this patient group are lacking. Here, the aim was to assess the changes in growth patterns before and after brain tumor diagnosis, the adult height, and the risk factors for compromised growth. The incidence and treatment practices of growth hormone deficiency were analyzed.

**Methods:**

A cohort of 73 survivors of childhood brain tumor (median age 27.2 years, range 16.2 to 43.8 years) was studied after a median follow-up period of 20.4 years from diagnosis (IQR 14.9 to 22.9 years). Patients were treated in five university hospitals in Finland between 1970 and 2008. Growth curves, final height, and patient- and disease-related risk factors for compromised growth during different growth periods were analyzed. Laboratory analyses for IGF-1 and IGFBP-3 were performed at the follow-up.

**Results:**

Growth failure was evident at diagnosis, with a mean height decline of -0.6 SDS (standard deviation score) from birth (95% CI -1.15 to -0.05). Mean height SDS decline after the diagnosis was -1.09 SDS (95%CI -1.51 to -0.66). At follow-up, 37% of the study subjects (27/73) had true short stature (height < -2 SDS). The mean height deficit corrected for target height was -1.9 SDS (95% CI -1.45 to -2.40). Growth failure was associated with the age at diagnosis, corticosteroid dose, radiotherapy modality and mean dose of irradiation in the thalamic area. Low IGF-1 level (below -2.0 SDS) was found in 32% (23/72), and untreated growth hormone deficiency in 40% (29/72) of the subjects.

**Conclusion:**

Longitudinal growth impairment was common in radiotherapy-treated survivors of childhood brain tumor, resulting in compromised adult height. Loss of growth potential was evident already at diagnosis and further accelerated by the treatments. At young adulthood, unrecognized growth hormone deficiency was common.

## Introduction

Malignant brain tumors are the most prevalent solid tumors in childhood [1]. With current 5-year survival rates of 74–80% in the Nordic countries, the number of childhood brain tumor survivors is growing, which highlights the impact of late effects related to brain tumors and their treatment [2]. The effects of the malignancy itself and therapies such as radiotherapy and chemotherapy predispose these survivors to late morbidity and compromised adult height [3]. As many as half of the survivors are found to have at least one endocrine system disorder post-treatment, with growth hormone deficiency (GHD) the most common disorder of the hypothalamic–pituitary axis after cranial radiotherapy. This often results in compromised adult height, especially when not treated accurately [4–7]. Previous studies have shown low adult height in long-term brain tumor survivors [8], but the knowledge of the disease’s impact on growth potential, especially before diagnosis, is more limited [9]. Still, longitudinal studies on growth patterns during different growth periods in this patient group are lacking. GHD is highly linked to severe comorbidities such as metabolic syndrome and cardiovascular disease [10, 11], but the knowledge of current growth hormone deficiency and treatment status in adult brain tumor survivors is sparse.

In the current study, we evaluated the effects of malignancy, chemo- and radiotherapy on growth patterns before and after tumor diagnosis, along with adult height in a national cohort of radiotherapy-treated, long-term survivors of childhood brain tumor. We also assessed the prevalence of adult short stature, GHD, and tumor and therapy related risk factors for growth failure in this patient group.

## Subjects and methods

### Study subjects

**Fig 1:**
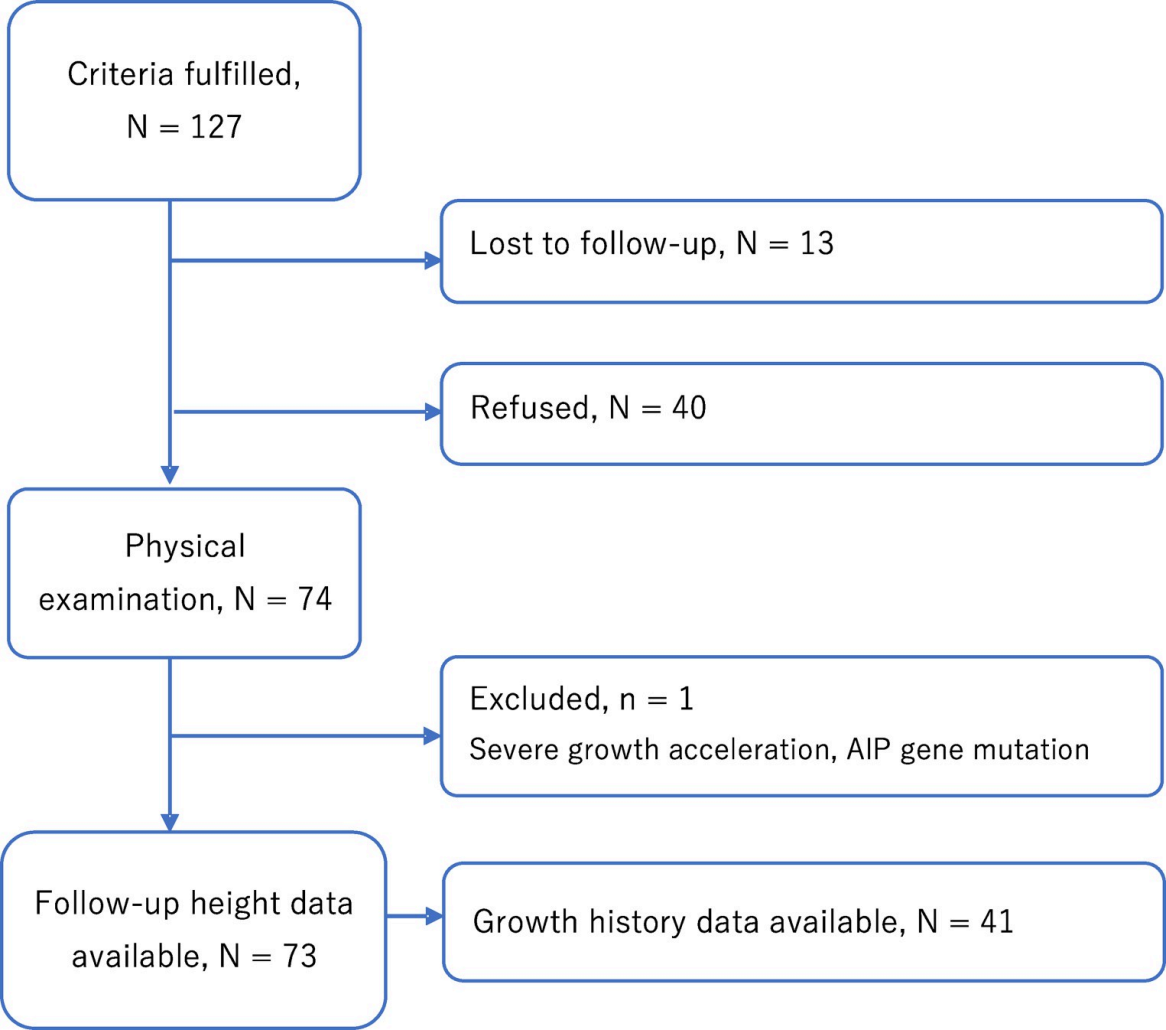
Flowchart of the cohort population.

This research is part of a cross-sectional national multi-center cohort study of radiotherapy-treated childhood brain tumor survivors in Finland. For this study, a cohort of radiotherapy-treated childhood brain tumor survivors were identified from the local registries of the five university hospitals in Finland (Oulu, Kuopio, Turku, Tampere, and Helsinki). A total of 127 subjects fulfilled the inclusion criteria and were invited to participate the study. Inclusion criteria included: diagnosis of the brain tumor before the age of 16 years, follow-up a minimum of 5 years after the cessation of brain tumor treatments, and minimum age of 16 years at the follow-up. Forty survivors declined to participate the study, and 13 (mean age 33.7 years, SD = 7.5 years, 47% females) were lost to follow-up. One male subject was excluded due the AIP3 gene mutation, leading to growth hormone excess and severe growth acceleration. A total of 73 subjects (46 male and 27 female, Fig 1) of the 127 eligible survivors (57%) treated between 1970 and 2008 participated in the study. The median follow-up period was 20.4 years (IQR 14.9 to 22.9 years); median age of the subjects at the end of the follow-up was 27.2 years (IQR 23.9 to 32.0 years). There were no significant differences between the non-participants and participants. Detailed cohort analysis is described in Remes et al. [12].

Clinical patient data, including information on growth at any time point from birth to follow-up, were gathered from the local patient registries of the five university hospitals. GHD after the cessation of the tumor treatments was confirmed by growth hormone stimulation tests done by local endocrinologists. A thorough physical examination at the time of the follow-up, including auxological measurements, was performed by a physician [12]. To evaluate potential growth hormone dysfunction in the patients, information on growth hormone therapy (GHT) at any time point was gathered from the patients’ files, and insulin-like growth factor 1 (IGF-1) or insulin-like growth hormone binding protein 3 (IGFBP-3) blood levels were measured at the follow-up. Information regarding current medications was gathered via a questionnaire that was filled during the follow-up visit.

### Laboratory analyses

Overnight fasting blood samples were collected between 7:30 AM and 10:00 AM and were analyzed in NordLab, Oulu University Hospital to study the levels of IGF-1 and IGFBP-3 at the follow-up. IGF-1 and IGFBP-3 were measured and available for 72 subjects. IGF-1 and IGFBP-3 were analyzed with an Immulite 1000 Immunoassay, Siemens Healthcare, Munich, Germany.

### Growth data

Height (74 subjects) was measured as part of a comprehensive physical examination using a calibrated stadiometer. Age- and gender-specific length/height standard deviation score (SDS) values at birth, at the time of diagnosis, and at the time of the follow-up examination (adult height) were calculated using Finnish growth references for children and adolescents aged 0 to 20 years [13, 14]. All subjects had achieved their adult height at the time of the examination. Growth data from the well child health clinics’ records were available for 42 subjects. Birth length data were available for 31 subjects, and parents’ height data for 29 subjects. Target height was calculated using a new Finnish growth reference model based on parental height data. One subject was excluded from the analysis because of having a growth hormone-secreting tumor of the pituitary gland. Total height deficit was calculated as the difference between adult height SDS and target height SDS. Untreated GHD at the follow-up was defined as an IGF-1 and/or IGFBP-3 Z-score below -2.0, independent of growth hormone treatment status at any point.

### Statistical methods

Changes in height SDS between different time points were analyzed. The SDS difference between the target height SDS and the adult height SDS at the end of the follow-up period was measured. Age- and gender-standardized Z-scores were calculated for IGF-1 and IGFBP-3 blood levels according to the immunoassay references. Results are expressed as mean (standard deviation, std) for normally distributed data and median (range) for skewed data, if not otherwise mentioned. Height and age data were normally distributed. Changes in length/height SDS over time (birth, diagnosis, follow-up) were analyzed with Linear mixed model (LMM), with random intercept and first-order autoregressive covariance structure for repeated measurements method with Sidak adjustments for post-hoc tests. The differences in the distributions between dichotomous variables were evaluated using a Chi2 test. One-way ANOVA analysis was used to evaluate mean differences between multiple groups (age and treatment modality), with Tukey’s HSD correction for post-hoc tests. Logistic regression was used to calculate odds ratio for dichotomous variables (radiotherapy modality, chemotherapy, number of hormone deficiencies, corticosteroid dose). P < 0.05 was considered statistically significant. All p-values were two-sided. For the analyses, we divided the subjects into different age groups according to the age at diagnosis, taking into consideration the different growth phases during childhood: age group 1 (0 to 6 years old; prepubertal), age group 2 (7 to 11 years old; peripubertal), and age group 3 (12 to 16 years old; pubertal) [15]. Due to the variability of the chemotherapy regiments, cyclophosphamide equivalent dose was calculated for quantifying alkylating agent exposure [16].

### Ethics

Written informed consent was obtained from all enrolled subjects and/or their legal guardians for the clinical study. The study was approved by the institutional Review Boards of Oulu, Kuopio, Turku, Tampere, and Helsinki university hospitals, Finland. This research has been provided with authorization from the ethics committee of the Northern Ostrobothnia Hospital District (16.9.2010/56/2010) and has been reported to the research register of Oulu University Hospital (25.8.2010/159/2010). The research was conducted according to the principles of the Declaration of Helsinki.

## Results

### Cohort characteristics

Clinical and demographic data of the entire study population are shown in Table 1. Almost half of the subjects (46%, n = 34) were diagnosed with brain tumor before the age of 7 years. In 52% (n = 38) of the subjects, the tumor was infratentorial. Tumors located in the diencephalon, potentially affecting the function of the hypothalamus pituitary function, were diagnosed in 27% (n = 20) of the subjects. Of the subjects, 59% (n = 43) had received cranial radiotherapy (CRT) and 41% (n = 30) had received craniospinal therapy (CSRT). Other brain tumor treatments included surgery and/or chemotherapy (Table 1). The majority of the subjects had undergone surgical operation, and over half had received chemotherapy. Of all the ventriculoperitoneal shunts (VPS), 45% (n = 20) and 11% (n = 5) were found in subjects with cerebellar tumors and tumors located in the fourth ventricle, respectively.

**Table 1:** Clinical and demographic data on the 73 childhood brain tumor survivors.

		*n*, *N = 73*	(%)
Gender	*Male*	46	62
	*Female*	27	37
Age at diagnosis, years	0–6	34	47
	7–11	22	30
	12–16	17	23
Location of tumor	*Hemispheric*	12	16
	*Diencephalon*	20	27
	*Midbrain*	1	1
	*Cerebellum*	26	36
	*Pons*	4	6
	*Intraventricular*	10	14
Operation	*Total resection*	29	39
	*Partial resection*	31	43
	*Tumor biopsy*	10	14
	*No operation*	3	4
Radiotherapy	*Local cranial*	38	52
	*Whole brain*	3	4
	*Craniospinal*	30	41
	*Stereotactic*	2	3
Chemotherapy	* *	47	64
Treatment combination	*Cranial*	23	32
	*Cranial with chemotherapy*	20	27
	*Craniospinal*	2	3
	*Craniospinal with chemotherapy*	28	38
Ventriculoperitoneal shunt		44	60
Number of hormonal dysfunctions in one patient	0	19	26
	1	16	22
	2	17	23
	3	15	21
	4	6	8
Growth hormone treated	* *	36	49

Number of patients and percent of the population or the subgroup are shown.

### Adult height

The growth data, including mean length/height SDS at birth, diagnosis, and follow-up, are shown in Table 2A and 2B. In the entire study population, adult height at the follow-up was 158 cm (-1.72 SDS) for females and 170 cm (-1.56 SDS) for males. Altogether, 37% (27/73) of the subjects had short stature; the final adult height was below -2 SDS. Adult short stature was most common among subjects with age at diagnosis below 7 years (70%, 19/27). Target height data were available for 28 subjects. In these, the mean growth difference between target height and adult height was -1.28 SDS (95% CI -0.38 to -1.17). In this group, growth loss of more than 1.0 SDS and 2.0 SDS was found in 57% (16/28) and 29% (8/28) of the subjects, respectively. The mean growth deficit was 1.17 SDS in males and 1.56 SDS in females, and the mean difference between genders was 0.40 SDS, 95% CI -0.48 to 1.27.

**Table 2:** Linear mixed model analysis with pre- (A) and post-diagnosis (B) growth predictors.

A										
*Pre-diagnosis predictors*	Birth(n = 30)			Diagnosis(n = 41)			Follow-up(n = 73)			Main effects	Time * Predictor	Birth vs.Diagnosis	Diagnosis vs Follow-up
	Mean	(SD)	Mean	(SD)	Mean	(SD)	p-value	p-value	95% CI	95% CI
***Age*, *years***										
*0–6*	-0.02	1.28	-0.71	0.98	-2.25	1.34	0.072	0.015	-0.106 to 1.447	**0.818 to 2.258** ^**♦**^
*7–11*	-0.1	0.49	-0.19	1.29	-1.4	1.18			-1.238 to 1.622	**0.268 to 2.159** ^**♦**^
*12–16*	-0.13	0.61	-0.58	1.22	-0.65	1.24			-0.529 to 1.492	-0.760 to 1.049
***Gender***										
*Male*	-0.25	0.83	-0.52	1.13	-1.72	1.24	0.825	0.459		
*Female*	0.25	1.27	-0.56	1.14	-1.56	1.68				
***Diencephalon tumor***										
*Yes*	0.39	1.53	-0.97	0.35	-1.49	1.61	0.612	0.333		
*No*	-0.23	0.81	-0.48	1.22	-1.67	1.38				
***Hypophysis tumor***										
*Yes*	-0.28	0.60	-0.90	0.39	-0.91	1.20	0.943	0.121		
*No*	-0.02	1.09	-0.49	1.19	-1.70	1.42				
**B**										
*Post- diagnosis predictors*			**Diagnosis (n = 41)**		**Follow-up(n = 73)**		**Main effects**	Time * Predictor interaction	**Diagnosis vs Follow-up**	
			Mean	(SD)	Mean	(SD)	p-value	p-value	95% CI	
***Corticosteroid dose*. *mg/m2***										
*< 5 000* mg*/m2*			-0.45	1.06	-1.29	1.27	0.157	0.011	**0.293 to 1.437** ^**♦**^	
*> 5 000* mg*/m2*			-0.81	1.32	-2.39	1.44			**0.592 to 2.513**	
***Radiotherapy***										
*Cranial*			-0.74	1.00	-1.53	1.55	0.766	0.056	**0.135 to 1.388** ^**♦**^	
*Craniospinal*			-0.14	1.27	-1.76	1.20			**0.843 to 2.485** ^**♦**^	
***Ventriculoperitoneal shunt***										
*Yes*			-0.40	1.26	-1.71	1.45	0.227	0.073		
*No*			-0.73	0.89	-1.48	1.37				
***Treatment combination***										
*Cranial no chemotherapy*			-0.67	1.18	-1.40	1.06	0.959	0.208		
*Cranial with chemotherapy*			-0.82	0.82	-1.68	1.99				
*Craniospinal no chemotherapy*			-0.55	0.64	-1.95	1.06				
*Craniospinal with chemotherapy*			-0.08	1.36	-1.74	1.23				
***Growth hormone substitution***										
*Yes*			-0.61	1.00	-1.91	1.43	0.228	0.530		
*No*			-0.48	1.25	-1.34	1.35				

Mean length/height SDS and standard deviation at birth, diagnosis, and follow-up are shown. Significant 95% confidential intervals for time*predictor interaction are flagged (**♦)**.

### Risk factors for adult short stature

Linear mixed model analyses were performed to investigate the influence of different factors on adult height SDS. Young age at diagnosis, high corticosteroid dose, and irradiation maximum of the thalamus area used during brain tumor treatments were associated with adult short stature. The mean difference was 1.07 SDS (p = 0.018, 95% CI 1.97 to 0.17) between age groups 1 and 2 and 1.53 SDS (p = 0.001, 95% CI 2.32 to 0.74) between age groups 1 and 3. Subjects treated with a total corticosteroid dose of more than 5000 mg/m2 experienced greater growth loss than those with a lower corticosteroid dose (mean difference 0.85 SDS, p = 0.043, 95% CI 0.03 to 1.67). Cyclophosphamide equivalent dose was not associated with the growth failure after the diagnosis (R = 0.206, p > 0.05).

The mean dose of irradiation in the thalamic area (ThIRD), available for 63 survivors, was 42.5Gy (SD = 14.3Gy). More severe growth decline from birth to the follow-up correlated with higher ThIRD (r = -0.411) also when controlling for GHT at any time point before the follow-up (r = -0.417). Growth decline was more significant when ThIRD exceeded 40Gy. The mean height difference between these ThIRD groups (below or above 40Gy) was 1.84 SDS (p = 0.015, 95% CI 3.28 to 0.39). For 15 subjects who received GHT at any time point, a more severe height deficit (corrected for target height) correlated with a longer time lapse for GHT initiation (r = 0.567).

### Height SDS loss prior to diagnosis

**Fig 2:**
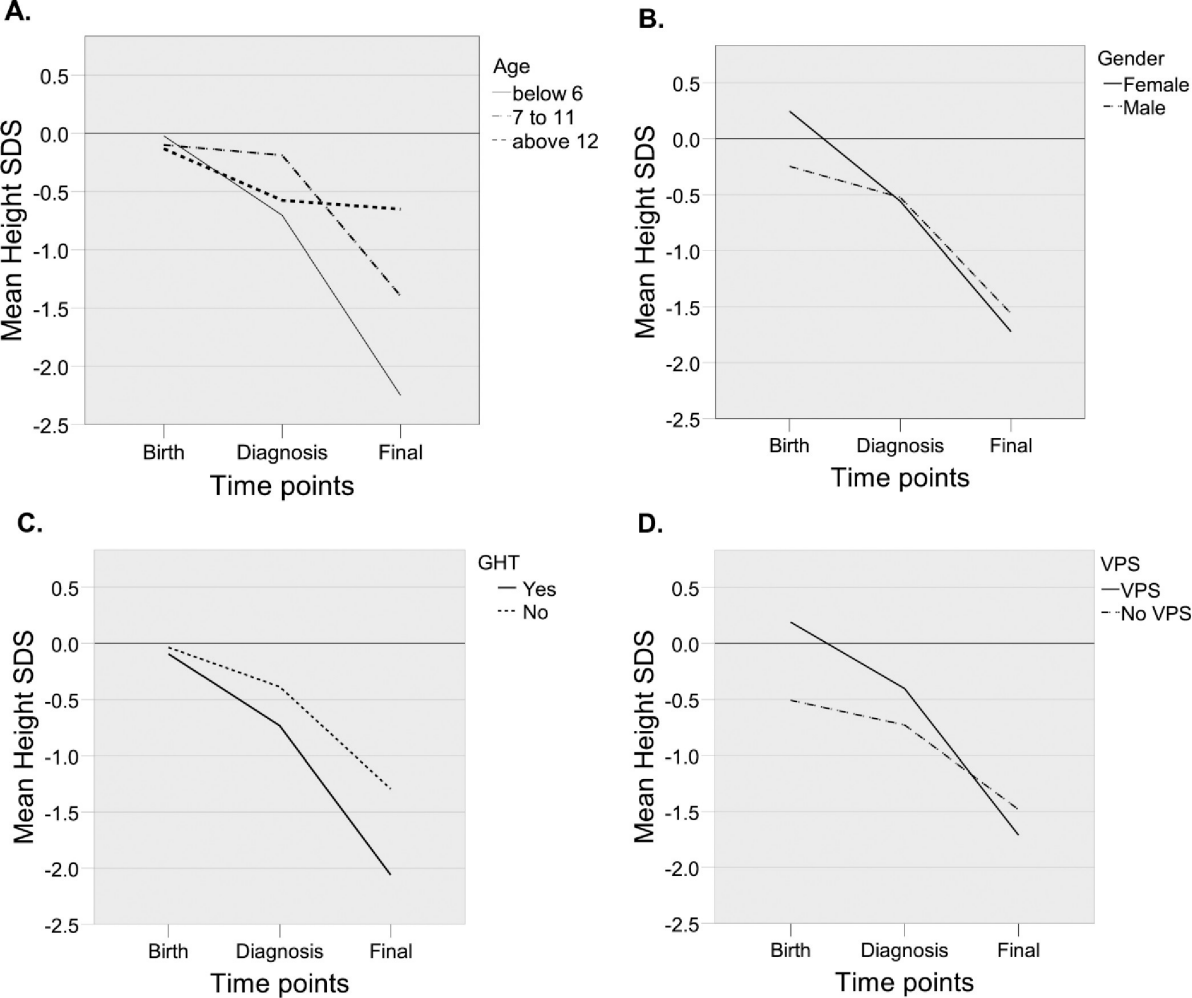
Mean length/height SDS at birth, diagnosis, and follow-up. By age (A), patient gender (B), and growth hormone replacement therapy (GHT) at any time (C) or ventriculoperitoneal shunt (VPS) (D).

The subjects were shorter at diagnosis when compared to the general population (mean -0.54 SDS, p = 0.004, 95% CI -0.89 to -0.18) (Fig 2, Table 2A). A significant change in height SDS was observed when comparing the length SDS at birth to the height SDS at diagnosis, with a mean of -0.6 SDS (p = 0.033, 95% CI -1.15 to -0.05). The height SDS decline at diagnosis was deepest among the youngest subjects, but the differences between the age groups were not significant (Table 2A, Fig 2A).

### Height SDS change from diagnosis to adult height

The height SDS declined further after diagnosis, with a significantly lower height SDS at adult height when compared to height SDS at diagnosis in both genders (Fig 2B, Table 2A). The mean height SDS decline in the study population was -1.09 SDS (p < 0.001, 95%CI -0.66 to -1.51). This growth loss was evident despite GHT (Fig 2C). To analyze the effects of patient- and treatment-related factors on growth changes over the whole growth period, a linear mixed model analysis (LMM) was performed. LMM showed a significant association between age at diagnosis, total corticosteroid dose, and changes in length/height SDS over time points from birth to the follow-up (Table 2A and 2B). There was an associative interaction effect of the irradiation modality on the height SDS changes over time points, with more severe height SDS decline from diagnosis to the follow-up in the subjects treated with craniospinal radiotherapy (Table 2B). The need for VPS did not correlate with adult height in our study population (Table 2B, Fig 2D).

### Effect of tumor on growth pattern

Subjects diagnosed with a tumor localized in the diencephalon or closely adjoining it (27% (20/73), including the locations: suprasellar region, sella turcica, hypophysis, optic chiasm or opticus, third ventricle) experienced mean growth decline of -0.98 SDS (95% CI -2.06 to 0.10) before diagnosis. Mean height decline after diagnosis was -0.14 SDS (95% CI -0.55 to 0.82). Adult height mean deficit of these patients was 0.70 SDS (p = 0.009, 95% CI 0.24 to 1.16).

Tumor distribution in our population is shown in supplement 1 (S1 Table). Analyses were performed for most prevalent tumor types; astrocytoma (32.9%) and medulloblastoma (26.0%). Prior to diagnosis, subjects with astrocytoma (middle brain 33% (8/24) and posterior fossa 46% (11/24)) experienced mean growth decline of -1.14 SDS (95% CI -2.94 to 0.67) and mean growth decline after diagnosis of -0.45 SDS (95% CI -1.31 to 0.40). Subjects with medulloblastoma (posterior fossa 100% (19/19)) had a mean growth decline as high as -2.34 SDS (p < 0.001, 95%CI -3.15 to -1.53) after diagnosis, resulting in an adult height deficit of 1.66 SDS (p = 0.021, 95%CI 0.41 to 2.91). Subjects with tumors located in the posterior fossa experienced significant growth decline after diagnosis (mean -1.53 SDS, p < 0.001, 95%CI -2.16 to -0.90) and, eventually, an adult height deficit of 1.45 SDS (p = 0.001, 95%CI 0.71 to 2.20).

### Growth hormone deficiency

Of the subjects, 49% (n = 36) had been diagnosed with GHD and had received GHT. Of those who had received GHT at any point, only 16% (6/36) had growth hormone replacement therapy at the follow-up visit. At the follow-up, 46.6% (34/73) of the subjects used thyroxine, of which 78.8% (26/34) of the treatments had been initiated before the age of 16 years. Of the female subjects, 46.4% (13/28) received estrogen, and 13.0% of the male subjects (6/45) received testosterone treatment. At the follow-up, 9.6% of the subjects (7/73) diagnosed with panhypopituitarism had hydrocortisone treatment. Earlier GHT strongly associated with a higher number of other hormonal dysfunctions in the same subject (mean difference 1.1, p < 0.001, 95%CI 0.56 to 1.66), mode of radiotherapy (OR 4.35 for CSRT vs. CRT, p = 0.004, 95%CI 1.60 to 11.90), total corticosteroid dose below vs. over 5000 mg/m^2^ (OR 18.00, p < 0.001, 95%CI 3.38 to 86.75), and chemotherapy (OR = 5.27, p = 0.003, 95% 1.78 to 15.67). Mean adult height of subjects who had received GHT was -0.57 SDS lower comparing to those with no GHT (95%CI -1.22 to 0.08) (Fig 2C).

**Fig 3:**
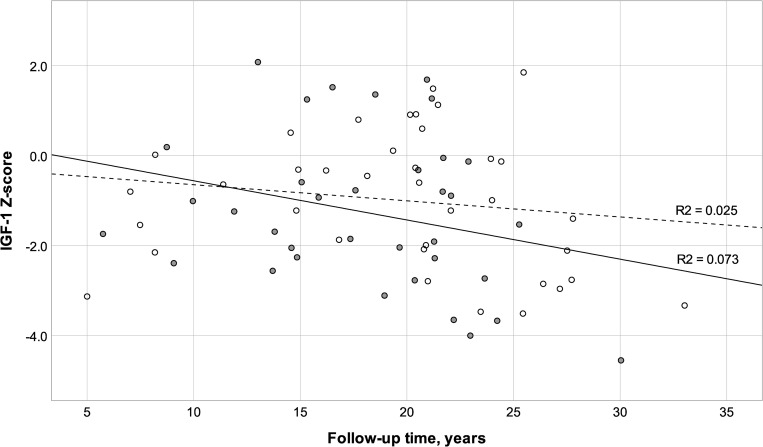
IGF-1 Z-score versus follow-up time of subjects who received growth hormone therapy (GHT) at any time point (solid dots) and those with no GHT (hollow dots).

The concentrations of IGF-1 and the Z-scores for IGF-1 and IGFBP-3 at follow-up are shown in Fig 3 and Table 3, respectively. Untreated GHD defined by IGF-1 and/or IGFBP-3 Z-score below -2.0 at the time of the follow-up was found in 40% (29/72) of the subjects, of which 45% (13/29) had not received GHT at all. Of these subjects, 55% (16/29) had been treated with growth hormone following tumor treatments, and 1 subject remained on GHT at the follow-up (age 21.6 years). The mean IGF-1 concentration in this group was 11.4 nmol/l (SD = 6.6 nmol/l, range 0.0 to 25.6 nmol/l). Growth hormone treatment initiation date was available for 31 subjects. Mean GHT lapse after tumor treatments in these subjects was 44 months (SD = 46 months, range 4–218 months). Mean GHT lapse among 14 subjects diagnosed with currently untreated GHD was 52 months (SD = 64 months) and for those whose IGF-1 was found to be normal 36 months (SD = 24 months). GHT lapse time was dependent on the age at diagnosis (r = -0.582) and correlated with more severe adult height deficit (r = 0.567). There was no correlation between GHT lapse time and IGF-1 or IGF-1 Z-score. Levels of IGF-1 (r = -0.346) correlated significantly with the follow-up time (Fig 3).

**Table 3:** Mean (SD) IGF-1 and IGFBP-3 Z-scores of 72 subjects at follow-up.

	*IGF-1*		*IGFBP-3*	
	Yes	No	Yes	No
**GHT at any time point**	-1.26 (1.71)	-0.99 (1.51)	-1.40 (1.27)	-1.43 (0.94)
*95%CI of mean difference*	-1.03 to 0.49		-0.50 to 0.55	
**GHT at follow-up**	0.60 (1.64)	-1.28 (1.52)	-0.80 (0.84)	-1.47 (1.11)
*95%CI of mean difference*	0.58 to 3.18 **		-0.26 to 1.61	
**Untreated GHD**	-2.56 (0.96)	-0.15 (1.16)	-2.23 (1.08)	-0.87 (0.72)
*95%CI of mean difference*	-2.93 to -1.90 **		-1.79 to -0.94 **	
**Ventriculoperitoneal shunt**	-0.82 (1.63)	-1.59 (1.01)	-1.36 (1.17)	-1.50 (1.01)
*95%CI of mean difference*	0.01 to 1.53 *		-0.39 to 0.68	
**Diencephalon tumor**	-1.47(2.06)	-0.99 (1.39)	-1.90 (1.46)	-1.23 (0.89)
*95%CI of mean difference*	-1.51 to 0.54		-1.39 to 0.05	

* Significant at p < 0,05

** significant at p < 0,01

GHD growth hormone deficiency, GHT growth hormone therapy

## Discussion

In the current study, the majority of the subjects in our cohort were found to have suffered loss of their growth potential. Altogether, 37% of our study subjects had short adult height when using the criteria of short stature being adult height below –2 SDS on gender- and population-specific growth charts [17]. Previous studies addressing the adult height in childhood tumor survivors clearly show the negative impact of the disease and the treatments on growth. Short stature (-2 SDS) prevalence varies between 8% and 46% among adult survivors of childhood brain tumor who received CRT or CSRT [18–20]. According to our results, the occurrence of short stature after radiotherapy-treated childhood brain tumor is high, and substantial loss in growth potential occurs even before diagnosis.

Studies on survivors of radiotherapy-treated childhood brain tumor with a focus on growth patterns prior to diagnosis are sparse. Height at diagnosis has been shown to be one of the most initial determinants for adult height among childhood central nervous system tumor survivors [18, 21], reflecting the impact of pre-diagnosis growth on adult height potential. We detected a significant decline in height SDS at diagnosis, compared with the general population, which suggests an important impact of the disease itself on growth and adult height. In previous studies, growth retardation prior to diagnosis of -0.6 up to -2 height SDS was shown among pre- and peripubertal patients diagnosed with tumor on the hypothalamic–pituitary axis [22–24]. Qi et al. showed growth decline years before diagnosis among craniopharyngioma patients [25]. In our study, the growth decline before diagnosis was more severe among subjects diagnosed with tumors located in the diencephalon region. The high prevalence of growth failure at diagnosis in our study cohort might be affected by a high prevalence of midline supratentorial tumors (29%) in contrast to the 15–20% in a previous study of Pollac et al [26].

The malignancy itself may affect the complex regulation of normal growth and development, resulting in poor growth [27]. Brain tissue is particularly vulnerable during the sensitive developmental period of childhood, and growth failure can be seen years before any other symptoms [28]. Santoro et al. reported that of the patients in their study, 9% with opticus glioma already had GHD at diagnosis. In their study, tumors involving the hypothalamus and located in the anterior optical tract region were likely to be diagnosed with an endocrine disorder [29]. We hypothesize that growth failure at diagnosis seen among patients with tumors located in the diencephalon and closely adjoining the anterior hypothalamic area may be due to the local dysfunction of the anterior hypophysis through tumor compression or inflammation, thus leading to GHD and growth retardation prior to diagnosis.

We detected a further decline in height SDS following treatments of the brain tumor. Negative effects of corticosteroid therapy during early childhood on linear growth have been recognized [30]. The most important determinants of growth loss after diagnosis were age at diagnosis, height SDS at diagnosis, high corticosteroid dose, CSRT, and tumor location. Patients with medulloblastoma diagnosis experienced steep growth loss after diagnosis, reflecting the severity of the disease and its treatments. The negative impact of these factors on growth has also been shown by others [9, 30–32].

In our study, the occurrence of GHD at any point was high, as 46% of the subjects suffered from GHD. We used IGF-1 level to diagnose GHD at follow-up, as earlier studies have shown that a low IGF-1 level reliably predicts GHD among survivors of radiotherapy-treated childhood brain tumor [33, 34]. In our study, the IGF-1 level was low in the majority of the study population and even undetectable in two subjects. To our surprise, untreated GHD at the follow-up was found in 40% of the subjects, of whom 45% had not received growth hormone treatment at all. Time lapse for the initiation of GHT varies due to individual factors and current treatment practices. In previous studies, no effect of the lag time for GHT initiation on the adult height was found [20]. Our results showed that the median time lapse between tumor treatment finalization and the initiation of GHT was 2.7 years (0.3 to 17.3 years), which is in line with other published studies [8]. We found that time lapse for GHT strongly correlated with age at diagnosis.

There has been a tendency toward earlier initiation of GHT in more recent years, supporting the more liberal treatment practices [8]. These numbers clearly show that this patient group is undertreated, possibly because of concern over the risk of recurrence or the occurrence of new malignancies. Risks of GHT in central nervous system tumors among pediatric and adult patients are still not fully clear [35]. Several recent studies have shown no association between GHT and new malignancies, either recurrence or secondary tumor, among childhood tumor survivors [36, 37]. Meningioma is a common secondary neoplasm after radiotherapy-treated brain tumor [38]. However, early GHT did not associate with the risk for meningiomas among late survivors of childhood brain tumor [36].

Previous studies have shown that in adults, GHD is highly linked to severe comorbidities such as metabolic syndrome and cardiovascular disease, demonstrating a clear indication and need for proper treatment of GHD [10, 39, 40]. Caicedo et al. suggested that GHT may play a beneficial role in the treatment and prognosis of cardiovascular events [11]. The psychosocial consequences of GHD and short stature in an otherwise healthy population have been studied extensively, but the results are largely controversial [41, 42]. GHD and short adult stature itself may have a profoundly negative impact on an individual’s well-being, specifically if there are other co-morbidities, that are common in radiotherapy-treated long-term survivors of childhood brain tumor [43]. However, height appears to have minimal consequences for physical functioning and negligible effects on other dimensions of quality of life in otherwise healthy adults [42, 43]. Still, early evaluation of growth restriction due to GHD and the initiation of GHT among childhood brain tumor patients is essential for achieving catch-up growth and preventing the comorbidities associated with GHD. It is important to recognize, that evaluation and treatment of GHD is still beneficial after achieving the adult height [44–46].

The strengths of our study were the long follow-up period and the clinical examination of the whole study group. Here, adult height was measured after the completion of the growth period, making the results reliable. In addition, the information of childhood growth prior to diagnosis of each subject was measured and gathered by medical personnel, making the data reliable. Our study population represents well the radiotherapy-treated childhood brain tumor survivors in Finland; the population is genetically homogenous and the study centers use similar treatment protocols, making our results generalizable. Limitations of our study were the lack of information on growth hormone status. Also, lack of complete growth data prior to the initiation of tumor treatments is a recognized limitation of our study. The target height was available only for a part of our study subjects, which limits the power of the study. Length measurement at birth has its own limitations and may vary depending on the measurement set-up. However, environmental and genetic factors are considered the most important determinants of linear growth after birth, rather than birth length alone [47].

## Conclusions

Growth is severely compromised among the majority of radiotherapy-treated long-term survivors childhood brain tumor. Growth decline is already evident at diagnosis and is associated with young age and tumor localization. Short stature and untreated GHD at adult height are common. Growth hormone status should be screened periodically to facilitate the timely initiation of GHT.

## Supporting information

S1 Table Distribution of the tumor type of the cohort subjects. (PDF)
