# Correction: Pre- and postdiagnosis growth failure, adult short stature, and untreated growth hormone deficiency in radiotherapy-treated long-term survivors of childhood brain tumor

**DOI:** 10.1371/journal.pone.0294792

**Published:** 2023-11-17

**Authors:** Julia Anttonen, Tiina Remes, Pekka Arikoski, Päivi Lähteenmäki, Mikko Arola, Arja Harila-Saari, Tuula Lönnqvist, Tytti Pokka, Pekka Riikonen, Kirsti Sirkiä, Heikki Rantala, Marja Ojaniemi

There are errors in the Funding section. The correct Funding statement is: MO Cancer Foundation Finland https://syopasaatio.fi MO University and University Hospital of Oulu (Tutkimusrahoitus) and Lasten syöpäsäätiö Väre http://vareensaatio.fi and Pohjois-Suomen terveydenhuollon tukisäätiö www.terttusaatio.fi and University and University Hospital of Oulu (Tutkimusrahoitus) TR Special State Grants for Health Research in the Department of Pediatrics and Adolescence, Oulu University Hospital, Finland; TR the Väre Foundation for Pediatric Cancer Research, Finland; TR the Foundation of Päivikki and Sakari Sohlberg, Finlan; TR the Foundation of Arvo and Lea Ylppö, Finland; TR the Foundation for Pediatric Research, Finland; TR the Foundation of Emil Aaltonen, Finland; HR and AHS the Cancer Society of Finland; TR the Foundation of Thelma Mäkikyrö, Finland); TR the Cancer Foundation of Northern Finland; TR the Aamu Finnish Childhood Cancer Foundation; TR the Foundation of Alma and K. A. Snellman, Finland, and TR the Foundation of Märta Donner, Finland. The funders had no role in study design, data collection and analysis, decision to publish, or preparation of the manuscript.
