# Supplementary material for: Pre- and postdiagnosis growth failure, adult short stature, and untreated growth hormone deficiency in radiotherapy-treated long-term survivors of childhood brain tumor
Source: PLoS One. 2022 Sep 6;17(9):e0274274. doi: 10.1371/journal.pone.0274274 (PMC9447887; doi:10.1371/journal.pone.0274274)
Supplement: S1 Table — (PDF) [file pone.0274274.s001.pdf]

S1. Tumor type distribution

Tumor histology	n	% (N = 73)
Ependymoma	8	11.0%
Astrocytoma	24	32.9%
Adenoma	1	1.4%
Germinoma	7	9.6%
PNET	1	1.4%
AT-RT	1	1.4%
Histology unknowed	4	5.5%
ependymoblastoma	1	1.4%
Craniopharyngeoma	1	1.4%
Oligodendroglioma	1	1.4%
Medulloblastoma	19	26.0%
RGNT	1	1.4%
Meningeoma	1	1.4%
Oligoastrocytoma	1	1.4%
Medulloepithelioma	1	1.4%
Plexus chorioideus carcinoma	1	1.4%
